# Racial/Ethnic and Socioeconomic Disparities in Mental Health in Arizona

**DOI:** 10.3389/fpubh.2015.00170

**Published:** 2015-07-03

**Authors:** Luis Arturo Valdez, Brent A. Langellier

**Affiliations:** ^1^Health Promotion Sciences, Mel and Enid Zuckerman College of Public Health, The University of Arizona, Tucson, AZ, USA

**Keywords:** Hispanic, disparities, drug use, mental health, nativity

## Abstract

**Background:**

Mental health issues are a rapidly increasing problem in the US. Little is known about mental health and healthcare among Arizona’s Hispanic population.

**Methods:**

We assess differences in mental health service need, mental health diagnoses, and illicit drug use among 7,578 White and Hispanic participants in the 2010 Arizona Health Survey.

**Results:**

Prevalence of mild, moderate, or severe psychological distress was negatively associated with SES among both Whites and Hispanics. Overall, Hispanics were less likely than Whites to have been diagnosed with a mental health condition; however, diagnosis rates were negatively associated with SES among both populations. Hispanics had considerably lower levels of lifetime illicit drug use than their White counterparts. Illicit drug use increased with SES among Hispanics but decreased with SES among Whites. After adjustment for relevant socio-demographic characteristics, multivariable linear regression suggested that Hispanics have significantly lower Kessler scores than Whites. These differences were largely explained by lower Kessler scores among non-English proficient Hispanics relative to English-speaking populations. Moreover, logistic regression suggests that Hispanics, the foreign born, and the non-English language proficient have lower odds of lifetime illicit drug use than Whites, the US born, and the English-language proficient, respectively.

**Conclusion:**

The unique social and political context in Arizona may have important but understudied effects on the physical and mental health of Hispanics. Our findings suggest mental health disparities between Arizona Whites and Hispanics, which should be addressed via culturally- and linguistically tailored mental health care. More observational and intervention research is necessary to better understand the relationship between race/ethnicity, socioeconomic status, healthcare, and mental health in Arizona.

## Introduction

Mental health issues are a rapidly increasing problem in the US. The US Department of Health and Human Services defines mental health conditions as characterized by persistent, abnormal alterations in thinking, mood, or behavior associated with distress and impaired functioning (1). Over 24% of the American population lives with a diagnosed mental health condition, and over 45% will experience at least one diagnosable condition in their lifetime (2).

Some adults who suffer from mental health conditions meet diagnostic criteria for serious mental illness (SMI). SMI refers to adults who currently or at any time during the past year have a diagnosable mental, behavioral, or emotional disorder that has resulted in *functional impairment that limits major life activities*. Major depression, bipolar disorder, and schizophrenia are among conditions that typically meet this definition. The annual costs associated with SMI are estimated to be in excess of $300 billion as of 2012, excluding the cost attributable to physical health co-morbidities (3). The National Survey on Drug Use and Health (NSDUH) found that there were estimated 9.6 million US adults aged 18 or older in the U.S. with a SMI in 2012. American Indian/Alaskan Natives (8.5%) suffer from the highest prevalence of SMI, followed by Hispanics (4.4%) Whites (4.2%), and African Americans (3.4%) (4).

The prevalence mental health conditions are comparable between Hispanic and Whites; however, 18% of Whites use mental health services compared to just 7% of Hispanics (4). Estimates suggest that over 11 million adults aged 18 or older have an unmet need for mental health care (4). Access and use of mental health services is related to household poverty, living in impoverished neighborhoods, and lack of insurance or sufficient money to pay for necessary services (5–9). However, while lack of economic resources are a factor, Ojeda et al. found that service use among Hispanics is also affected by social barriers (e.g., stigma) (10). Furthermore, Hispanics that live in poor neighborhoods have less access to mental health services than their White counterparts (5, 11). Nevertheless, even when services are readily available, they often are not culturally or linguistically appropriate (6), and Hispanic patients are less likely to obtain adequate care when compared to their non-Hispanic counterparts (12). This phenomenon has been attributed to unavailability of Spanish language services (13), the lack of interpreters (6), scarcity of culturally competent service providers (6), and perceived discrimination (11). While many studies have found that those who do not speak English have lower probabilities of receiving needed services, an even more dramatic relationship was found between non-English speaking Hispanics and English-only Hispanics (14). Moreover, an even greater gap was found between non-English speaking Hispanic immigrants compared to US-born English-speaking Hispanics (15).

Hispanics make up 16.9% of the national population and 30.2% of the population of the state of Arizona (16). Projections suggest that the Hispanic population will double in the next 40 years and that by 2050 one in every three people living in the US will be Hispanic (16, 17). The large growth of the Hispanic population, coupled with its disproportionate burden of mental health issues and poor access to mental health services, underscores the need to identify proactive, comprehensive solutions. Thus, it is critical to understand the prevalence of diagnosed and undiagnosed mental health conditions, and access and use of mental health services among Hispanics. Because of the dire effects of mental health conditions, a first step toward developing effective strategies to improve mental health services among Hispanics is to further understand the extent and nature of disparities faced by this population. Moreover, it is imperative that the health of the Hispanic population in the state of Arizona, specifically, is further explored due explicitly to the sociopolitical implications of its proximity to the US–Mexico border, which have been found to have a detrimental impact on mental and physical health outcomes (18, 19).

In this study, our objectives are to (1) assess need, access, and utilization of mental health services as well as illicit drug use among Hispanic and White adults in Arizona, (2) assess whether disparities in mental health and illicit drug use are explained by socio-demographic, acculturative, and economic differences between Hispanic and White adults in Arizona.

### Conceptual framework

**Figure 1:**
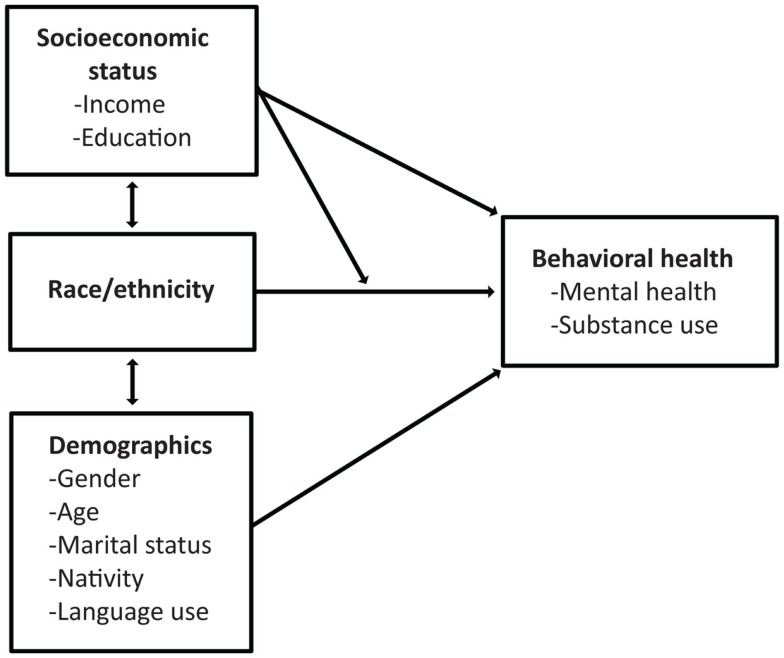
**Conceptual framework**.

We present the conceptual framework that guides our analyses in Figure 1. As per previous studies (4–15) we posit that there is a relationship between race/ethnicity and mental health. Specifically, literature suggests that there are Hispanic–White disparities in need, access, and use of mental health services and substance abuse (4–6, 8–15). Since previous studies have documented a relationship between age, marital status, and behavioral health (1–3), we further posit that a portion of these disparities can be explained by demographic differences between the Hispanic and White populations. Furthermore, the acculturation literature has documented different risk profiles between US-born, more acculturated Hispanics and their foreign-born, less acculturated counterparts (20–25). Therefore, it is also important to understand whether Hispanic–White disparities are explained by nativity and factors related to acculturation (e.g., language use). We further posit that the comparatively lower socioeconomic status of Hispanics places them at increased risk of behavioral health issues relative to Whites. Our final (exploratory) hypothesis is that socioeconomic status may moderate the relationship between race/ethnicity and behavioral health. Specifically, we believe that Hispanics’ behavioral health risk will vary based on SES through a number of potential (but unspecified) pathways. For example, low-income Hispanics frequently live in “ethnic enclaves” with high levels of social cohesion and social support, which may act as a buffer against a range of negative physical and mental health consequences (26, 27). Literature also shows differences in the benefits of educational attainment based on race/ethnicity, specifically showing that African Americans and Hispanics may see greater benefits from educational attainment that non-Hispanic Whites (28, 29).

## Materials and Methods

### Data source

Data for this study are from the second wave of the Arizona Health Survey (AHS), conducted from May through August of 2010. The AHS was designed to collect data on a range of indicators, including physical and mental health status, health-related behaviors, insurance coverage, and access to services. In brief the AHS was a population-based, list-assisted random-digit-dial telephone survey representative of the Arizona population living in households (30). Researchers selected residential telephone numbers from six geographic strata defined by counties or groups of counties. One adult respondent (age 18 and over) was selected from each household (30). In households with children (age 6 and under), one child was randomly selected and the adult most knowledgeable about the child’s health completed a child interview (30). Samples were selected to obtain at least 8,100 (although 8,215 were collected) adult interviews and 2,000 child interviews (30). AHS data are de-identified and publicly available. Secondary analysis of these data therefore does not constitute human subjects research as defined by federal regulations and does not require IRB review. Further details regarding the AHS study design and data collection are available elsewhere (30).

### Sample

The 2010 AHS included 8,215 adult respondents (30). For this study, we restricted our analyses to 7,578 White (*n* = 6,022) and Hispanic (*n* = 1,556) participants. Black or African American (*n* = 216), Asian/Pacific Islander (*n* = 99), Native American/American Indian (*n* = 243), and a small fraction (*n* = 79) of participants who refused to divulge race/ethnicity information were not included because our primary aim was to assess Hispanic–White disparities in mental health and drug use. Additionally, we further restricted our multivariable analyses to the subsample of participants with complete information about age, marital status, income, education, illicit drug use, mental health diagnosis, nativity, and English-language proficiency resulting in a multivariable analytic sample of *n* = 6,503.

### Measures

We assessed mental health service need using the Kessler K6 measure of psychological distress. The K6 has been validated for identification of current mental health problems and need for treatment (31, 32). The K6 consists of six questions about anxiety and depressive symptoms that a person has experienced in the most recent 30-day period (31, 32). For example, “During the past 30 days, about how often did you feel hopeless?” Each question is assessed on the following scale: 0 = none of the time, 1 = a little of the time, 2 = some of the time, 3 = most of the time, 4 = all of the time. All six questions are mandatory and total response scores can range from 0 (indicating no distress) through 24 (indicating severe distress) (31, 32). According to K6 diagnostic criteria, participants scoring from 0–6 (low range) are likely to be healthy but may benefit from early education-based prevention in order to prevent future mental health issues (31, 32). Participants that score 7–14 (mid range) are likely to have mild to moderate mental health disorders and are encouraged to access information and self-help treatment programs (31, 32). Participants that score between 15 and 24 (high range) are likely to have a severe mental health disorder and are encouraged to seek immediate help from a mental health professional (31, 32). We assessed mental health diagnoses based on participant self-report of diagnosis of bipolar or manic-depressive disorder, anxiety disorder, and clinical depression rates. Illicit drug use was assessed through self-reported lifetime illicit drug use, illicit drug use in the previous 12 months, and in the last 30 days. However, 12-month and 30-day drug use data yield insufficient power to examine between-group disparities, because if participants reported to never have used drugs, omitted questions about more recent drug created a skip pattern that resulted in a small prevalence of self-reported recent drug use. As a result, we elected to use lifetime drug use data. Marijuana, cocaine, crack cocaine, heroin, and methamphetamines were considered illicit drugs for this analysis. We categorized participants’ race/ethnicity as Hispanic or White. Federal Poverty Limit (FPL) is a combined measure of household income and family/household size (30). Federal poverty guidelines stipulate that a family of four living on a household income under $24,250 lives in a state of poverty (33). FPL was defined based on the following categories present in the AHS data file: <100% FPL, 100–199% FPL, 200–299% FPL, and ≥300% FPL. We categorized marital status as married, single, and widowed/separated/divorced. We separated educational attainment into less than high school, high school/equivalent, and more than high school. We also categorized English language proficiency as (1) native speaker or speaks very well, (2) speaks well, (3) not well, and (4) not at all. Lastly, nativity was categorized into US born and foreign born.

### Statistical analyses

We present means and 95% confidence intervals of all continuous variables and the percentage distribution of all categorical variables. We use conditional means and cross-tabulation to assess whether mental health outcomes and illicit drug use vary by race/ethnicity (i.e., Hispanic vs. White). We use t-tests to assess the statistical significance of variation across groups. We use multivariable regression models to examine the relationship between race/ethnicity and behavioral health outcomes. For each outcome (i.e., Kessler K6 scores and self-reported lifetime drug use), we present a series of four regression models. Consistent with our conceptual framework, we conduct “stepwise” analyses whereby additional sets of explanatory variables are added to each subsequent model. In the first model for each outcome, we adjust for race/ethnicity and demographic factors that vary between Hispanics and Whites (i.e., gender, age, and marital status). In the second model, we further adjust for socioeconomic characteristics (i.e., household income, measured as a percentage of FPL, and educational attainment). In the third model, we further adjust for English language proficiency and nativity to understand the extent to which Hispanic–White disparities that remain after adjustment for other factors may be attributable to nativity and acculturation. In the fourth model, we include an interaction between race/ethnicity and household income to assess whether Hispanics’ level of behavioral health risk varies across income strata. In the final model, we include an interaction between race/ethnicity and educational attainment to assess whether Hispanic’s level of behavioral health risk varies by educational attainment level.

The outcome in the multivariable linear regression models is the square root of the Kessler K6 score. We use the square root because the K6 is right tailed and violates the normality assumption of linear regression; using the square root transformation results in a more normally distributed outcome. We elected not to use the 12-month and 30-day drug use data because the number of self-reported drug use in the last 12 months and 30 days, respectively, yields insufficient power to examine between-group disparities. To account for non-response, probability of selection, and the complex survey design, we used weights present in the AHS data file. All data analyses were conducted using STATA 13 (34).

## Results

Table 1 contains demographic characteristics for the 1,480 Hispanic and 4,590 White participants in the sample. Hispanic respondents were significantly (*p* < 0.001) younger than White respondents. Over 75% of Hispanic respondents were under the age of 50 compared to 51% of Whites. Nearly 25% of Hispanic respondents were single compared to 15% of Whites (*p* = 0.002). Only 10% of Whites lived below the 100% FPL compared to 33% of Hispanics (*p* < 0.001). Over 55% of Whites lived above the 300% FPL while only 21% of Hispanics fell into the same category (*p* < 0.001). Approximately 95% of Whites were born in the US compared to 44% of Hispanics (*p* < 0.001). Nearly all Whites considered English to be their primary language compared to only 36% of Hispanics (*p* < 0.001). Finally while nearly all Whites were native speakers or speak English *very well*, only 49% of Hispanic respondents reported that they speak English *very well* (*p* < 0.001). No significant differences were found in the gender distribution of the two populations.

**Table 1:** **Demographic characteristics, mental health indicators, diagnosis rates, and illicit drug use of White and Hispanic respondents of the 2010 Arizona health survey (*n* = 6,070)**.

	White		Hispanic		*p*
	%	95% CI	%	95% CI	
**Gender**					0.782
Female	48.93	[46.52, 51.34]	49.68	[44.95, 54.42]	
Male	51.07	[48.66, 53.48]	50.32	[45.58, 55.05]	
**Age**					<0.001
<39	33.77	[31.07, 36.58]	52.62	[47.95, 57.24]	
40–49	17.43	[15.73, 19.26]	22.48	[19.11, 26.20]	
50–59	18.48	[16.97, 20.09]	14.52	[11.98, 17.50]	
60–69	15.13	[13.96, 16.38]	6.10	[4.73, 7.85]	
70–79	9.10	[8.34, 9.93]	3.38	[2.60, 4.39]	
80+	6.09	[5.44, 6.81]	0.89	[0.50, 1.43]	
**Marital**					0.002
Single	15.40	[13.18, 17.91]	19.96	[15.99, 24.63]	
Married	67.38	[64.92, 69.74]	69.29	[64.60, 73.62]	
Wid/div/sep	17.23	[15.81, 18.74]	10.74	[8.61, 13.33]	
**Living in poverty**					<0.001
Below 100% FPL	10.58	[8.90, 12.53]	33.47	[29.16, 38.07]	
100–200% FPL	16.70	[14.99, 18.57]	28.74	[24.47, 33.41]	
200–300% FPL	16.89	[15.20, 18.73]	15.90	[12.53, 19.97]	
More than 300%	55.82	[53.38, 58.24]	21.90	[18.56, 25.65]	
**US-born**	94.58	[93.27, 95.65]	44.24	[39.70, 48.88]	<0.001
**Primary language**					<0.001
English	96.93	[95.83, 97.75]	36.31	[32.08, 40.75]	
Spanish	1.02	[0.64, 1.62]	63.15	[58.69, 67.41]	
**English language prof.**					<0.001
Native/very well	98.53	[97.62, 99.10]	49.54	[44.81, 54.27]	
Well	0.46	[0.24, 0.85]	14.12	[11.15, 17.73]	
Not well	0.68	[0.28, 1.61]	24.45	[20.36, 29.05]	
Not at all	0.34	[0.14, 0.78]	11.89	[8.81, 15.87]	
**Kessler K6**					0.022
Low	75.68	[73.47, 77.76]	69.73	[65.15, 73.94]	
Mild/moderate	20.83	[18.84, 22.97]	26.46	[22.35, 31.02]	
Severe	3.49	[2.76, 4.41]	3.81	[2.74, 5.28]	
**Diagnosed mental health condition**					0.014
Diagnosed	17.97	[16.24, 19.84]	13.28	[10.56, 16.58]	
**Last illicit drug use**					<0.001
Never	64.97	[62.52, 67.34]	80.10	[76.33, 83.40]	
>12 Months	29.31	[27.07, 31.65]	15.59	[12.78, 18.88]	
Within last year	3.02	[2.17, 4.20]	1.39	[0.74, 2.58]	
Within last 30 days	2.70	[1.92, 3.79]	2.92	[1.52, 5.56]	
**Sample size**	6,022		1,556		

Table 1 also includes measures of psychological distress, mental health diagnosis, and self-reported lifetime use of illegal substances. There were differences in calculated K6 scores, diagnosis rates, and illicit drug use between Hispanics and Whites (*p* = 0.022). Nearly 76% of Whites reported a low K6 score compared to 69% of Hispanics. The mild/moderate/severe K6 score was also higher for Hispanics at 26% compared to 20% for Whites. *Severe* K6 score was similar for both populations. Hispanics had significantly lower rates of mental health diagnosis at 13% compared to Whites at nearly 18% (*p* = 0.014). Hispanics had lower rates of lifetime illicit drug use than Whites (*p* < 0.001). Hispanics also had lower rates of illicit drug use in the last year at 15% compared to nearly 30%. However, Hispanics had slightly higher use within the last month compared to Whites (*p* < 0.001).

Table 2 shows measures for psychological distress, mental health diagnosis and self-reported lifetime use of illegal substances stratified by FPL. When stratified by FPL, prevalence of K6 scores indicating mild/moderate/severe psychological distress decreased along with increased FPL for both populations. Hispanics improved from 38.74% when under the 100% FPL to 19.87% at or above 300% FPL (*p* = 0.003). Whites improved from 42.64% at under the 100% FPL to 16.89% when living at or above 300% FPL (*p* < 0.001). While Hispanics had lower rates of mental health diagnosis across all FPL levels when compared to Whites, diagnosis decreased along with increased FPL for both populations. Diagnosis rates for Hispanics decreased by nearly half, rising from 15% when living below the 100% FPL to 8% when living at or above the 300% FPL (*p* = 0.103). Similar trends are seen for Whites that went from nearly 29% while under 100% FPL to 14% at or above 300% FPL (*p* < 0.001). When living under the 100% FPL Hispanics have considerably lower levels of lifetime illicit drug use than their White counterparts at 8% compared to nearly 42%. However, illicit drug use levels change along with FPL. Hispanics show an increase from 8% at <100% FPL to 36% (*p* < 0.001) when living at or above the 300% FPL, while Whites decreased from nearly 42% at <100% FPL to 35% (*p* = 0.205) at 300%+.

**Table 2:** **Kessler score, mental health diagnosis, and illicit drug use by race/ethnicity and federal poverty limit (FPL) of White and Hispanic respondents of the 2010 Arizona health survey (*n* = 6,070)**.

Hispanic/White	FPL	Kessler K6 score mild/mod/severe		*p*	Diagnosed with any behavioral health condition		*p*	Ever use Illicit drugs		*P*
		%	[95% CI]		%	95% CI		%	95% CI	
**Hispanic**	<100%	38.74	[31.16, 46.91]	Ref.	15.49	[10.64, 22.02]	Ref.	8.45	[5.57, 12.60]	Ref.
	100–199%	29.92	[22.16, 39.03]	0.143	11.93	[7.82, 17.79]	0.351	15.92	[10.87, 22.74]	0.025
	200–299%	27.41	[17.95, 39.47]	0.144	18.44	[11.28, 28.68]	0.568	28.63	[18.87, 40.89]	<0.001
	300%+	19.87	[12.88, 29.36]	0.003	7.99	[3.80, 16.01]	0.103	36.26	[28.07, 45.33]	<0.001
**White**	<100%	42.64	[33.92, 51.84]	Ref.	28.98	[22.33, 36.67]	Ref.	41.94	[32.80, 51.68]	Ref.
	100–199%	34.79	[29.28, 40.75]	0.148	20.59	[16.69, 25.13]	0.041	30.50	[25.27, 36.29]	0.038
	200–299%	27.09	[22.25, 32.55]	0.003	21.42	[17.12, 26.44]	0.076	34.33	[28.72, 40.41]	0.178
	300%	16.89	[14.63, 19.41]	<0.001	14.06	[11.94, 16.48]	<0.001	35.60	[32.63, 38.69]	0.205

Table 3 includes the results of five multivariable linear regression models that assess the relationship between race/ethnicity and the square root of the Kessler score after adjustment for gender, age, and marital status (Model 1), adjustment for family income and educational attainment (Model 2), further adjustment for nativity and English language proficiency (Model 3). The fourth model includes interaction terms between race/ethnicity and household income. The final model includes interaction terms between race/ethnicity and educational attainment. Model 1 suggests that after adjustment for gender, age, and marital status, there is no significant relationship between race/ethnicity and Kessler score. After further adjustment for their comparatively lower levels of income and education, however, Model 2 indicates that Hispanics have significantly lower Kessler scores than Whites (*p* < 0.01). Model 3 suggests that lower Kessler scores among Hispanics could be explained by differences in English language proficiency between Hispanics and Whites. The fourth model reveals that Kessler score decreases with age (*p* < 0.001), is greater among the widowed/divorced/separated than among the married (*p* < 0.001), and decreases with household income (*p* < 0.001) and participants’ educational attainment (*p* < 0.01). However the fourth model does not reveal a significant effect of the interaction between race/ethnicity and family income. Moreover, the final model does not reveal a significant effect of the interaction between race/ethnicity and educational attainment. Finally, we assessed the *variance inflation factor* to test for variables that may introduce multicollinearity (i.e., English language proficiency, nativity) and found that all variance inflation factors were below 2.30 and were not a cause for concern.

**Table 3:** **Linear regression models predicting the square root of the Kessler K6 score of Hispanic and White adults in the 2010 AHS (*n* = 6,070)**.

	(1) b [95% CI]	(2) b [95% CI]	(3) b [95% CI]	(4) b [95% CI]	(5) b [95% CI]
**Race/ethnicity**					
White	Ref.	Ref.	Ref.	Ref.	Ref.
Hispanic	0.0205	−0.128**	−0.0357	0.049	−0.186
	[−0.0681, 0.109]	[−0.229, −0.0263]	[−0.142, 0.070]	[−0.194, 0.293]	[−0.419, 0.048]
**Female**	0.0636	0.057	0.0579	0.057	0.063
	[−0.004, 0.131]	[−0.009, 0.124]	[−0.008, 0.123]	[−0.008, 0.122]	[−0.003, 0.127]
**Age (y)**	−0.0094***	−0.00887***	−0.0087***	−0.009***	−0.009***
	[−0.0112, −0.0073]	[−0.0109, −0.0067]	[−0.011, −0.0066]	[−0.011, −0.007]	[−0.011, −0.006]
Married	Ref.	Ref.	Ref.	Ref.	Ref.
Single	0.0083	−0.0213	−0.0401	−0.038	−0.044
	[−0.104, 0.121]	[−0.136, 0.094]	[−0.153, 0.073]	[−0.15, 0.073]	[−0.156, 0.068]
Wid/div/sep	0.310***	0.228***	0.205***	0.204***	0.201***
	[0.214, 0.406]	[0.132, 0.322]	[0.109, 0.299]	[0.109, 0.299]	[0.107, 0.295]
**Household income (% FPL)**					
≤100%		Ref.	Ref.	Ref.	Ref.
101–200%		−0.0927	−0.116	−0.075	−0.108
		[−0.233, −0.048]	[−0.255, −0.023]	[−0.261, 0.11]	[−0.245, −0.290]
201–300%		−0.113	−0.159*	−0.115	−0.156*
		[−0.272, −0.045]	[−0.317, −0.016]	[−0.308, −0.078]	[−0.310, −0.002]
>300%		−0.318***	−0.361***	−0.322***	−0.353***
		[−0.457, −0.179]	[−0.501, −0.221]	[−0.494, −0.15]	[−0.49, −0.215]
**Education**					
<High school		Ref.	Ref.	Ref.	Ref.
High school		−0.0872	−0.164*	−0.166*	−0.276**
		[−0.235, 0.0602]	[−0.310, 0.018]	[−0.311, −0.021]	[−0.481, 0.015]
>High school		−0.158*	−0.236**	−0.239***	−0.313**
		[−0.302, −0.0134]	[−0.381, −0.091]	[−0.383, −0.094]	[−0.500, −0.118]
**Foreign born**			−0.091	−0.091	−0.09
			[−0.229, −0.046]	[−0.218, 0.183]	[−0.227, 0.071]
**English language**					
Native/very well			Ref.	Ref.	Ref.
well			−0.01	−0.025	−0.019
			[−0.215, −0.195]	[−0.133, 0.183]	[−0.227, 0.188]
Not well			−0.14	−0.163	−0.122
			[−0.364, 0.083]	[−0.389, 0.064]	[−0.342, 0.098]
Not at all			−0.39**	−0.418**	−0.342**
			[−0.655, −0.126]	[−0.686, −0.149]	[−0.613, −0.071]
**Interaction terms**					
Hispanic* 101–200%				−0.088	
				[−0.356, 0.180]	
Hispanic* 201–300%				−0.115	
				[−0.426, 0.194]	
Hispanic* >300%				−0.107	
				[−0.382, 0.167]	
Hispanic* high school					0.258
					[−0.015, 0.531]
Hispanic* >high school					0.132
					[−0.127, 0.392]
**Constant**	2.43***	2.77***	2.877***	2.848***	2.94***
	[2.286, 2.583]	[2.55, 2.99]	[2.667, 3.089]	[2.602, 3.09]	[2.696, 3.196]

95% confidence intervals in brackets. *p < 0.05; **p < 0.01; ***p < 0.001.

Table 4 includes the results of five multivariable logistic regression models that assess the relationship between race/ethnicity and lifetime illicit drug use. The first three models include the same sets of covariates as presented in the previous table. The fourth model adjusts for all covariates, but also includes interaction terms to assess whether the relationship between household income and illicit drug use varies between Hispanics and Whites. The fifth model also adjusts for all covariates, and includes interaction terms to assess whether the relationship between educational attainment and illicit drug use varies between Hispanics and Whites. The results presented in Model 1 suggest that, after adjustment for gender, age, and marital status, Hispanics are much less likely than Whites to have used illicit substances in their lifetimes. This relationship endures after further adjustment for income and educational attainment (Model 2), but is attenuated after further adjustment for nativity and English language proficiency (Model 3). However, adjustment in model 3 suggests that Hispanics (*p* < 0.05) the foreign-born, (*p* < 0.001), and the non-English proficient (*p* < 0.001) have lower odds of illicit drug use when compared to Whites, the US-born, and the English language proficient, respectively. Interestingly, the household income odds ratios in Model 4 suggest that there is no significant relationship between household income and illicit drug use among Whites; however, the interaction terms suggest that illicit drug use increases with household income among Hispanics. For example, Hispanics with annual household income >300% FPL have nearly three times the odds of having ever used illicit substances relative to those with income ≤100% FPL (*p* < 0.001). The final model does not uncover a significant effect of the interaction between race/ethnicity and educational attainment. Finally, we also assessed the variance inflation factor to test for variables that may introduce multicollinearity (i.e., English language proficiency, nativity) and found that all variance inflation factors were below 2.31 and were not a cause for concern.

**Table 4:** **Logistic regression models predicting lifetime illicit drug use among Hispanic and White adults in the 2010 AHS**.

	(1) OR [95% CI]	(2) OR [95% CI]	(3) OR [95% CI]	(4) OR [95% CI]	(5) OR [95% CI]
**Race/ethnicity**					
White	Ref.	Ref.	Ref.	Ref.	Ref.
Hispanic	0.359***	0.399***	0.786	0.331***	0.444*
	[0.275, 0.469]	[0.293, 0.544]	[0.581, 1.06]	[0.169, 0.649]	[0.222, 0.888]
**Female**	0.612***	0.623***	0.613***	0.615***	0.616***
	[0.504, 0.743]	[0.513, 0.758]	[0.504, 0.746]	[0.506, 0.747]	[0.507, 0.748]
**Age**	0.972***	0.971***	0.972***	0.971***	0.971***
	[0.966, 0.978]	[0.964, 0.977]	[0.966, 0.978]	[0.965, 0.9778]	[0.966, 0.977]
**Marital status**					
Married	Ref.	Ref.	Ref.	Ref.	Ref.
Single	0.731	0.746	0.655*	0.655*	0.649*
	[0.511, 1.045]	[0.515, 1.07]	[0.455, 0.945]	[0.455, 0.941]	[0.451, 0.933]
Wid/div/sep	1.271*	1.391**	1.234	1.235	1.217
	[1.001, 1.601]	[1.109, 1.77]	[0.981, 1.573]	[0.964, 1.582]	[0.950, 1.561]
**Household income (% FPL)**					
≤100%		Ref.	Ref.	Ref.	Ref.
101–200%		1.067	0.95	0.749	0.963
		[0.679, 1.369]	[0.635, 1.42]	[0.466, 1.204]	[0.642, 1.444]
201–300%		1.476*	1.13	0.872	1.131
		[0.926, 1.918]	[0.754, 1.69]	[0.545, 1.395]	[0.756, 1.689]
>300%		1.627**	1.27	0.99	1.277
		[1.040, 2.36]	[0.871, 1.697]	[0.649, 1.52]	[0.878, 1.858]
**Education**					
<High school		Ref.	Ref.	Ref.	Ref.
High school		1.032	0.685	0.693*	0.564*
		[0.696, 1.356]	[0.453, 1.035]	[0.458, 1.047]	[0.340, 0.934]
>High school		0.909	0.604**	0.609**	0.498**
		[0.611, 1.35]	[0.404, 0.903]	[0.409, 0.908]	[0.306, 0.81]
**Foreign born**			0.388***	0.392***	0.387***
			[0.243, 0.619]	[0.245, 0.625]	[0.243, 0.615]
**English language**					
Native/very well			Ref.	Ref.	Ref.
Well			0.537	0.624	0.583
			[0.257, 1.126]	[0.296, 1.32]	[0.285, 1.189]
Not well			0.212**	0.278**	0.258*
			[0.078, 0.575]	[0.099, 0.734]	[0.094, 0.701]
Not at all			0.02***	0.026***	0.027***
			[0.005, 0.099]	[0.006, 0.13]	[0.006, 0.128]
**Interaction terms**					
Hispanic* 101–200%				2.417*	
				[1.101.5.73]	
Hispanic* 201–300%				2.815**	
				[1.214, 6.526]	
Hispanic* >300%				2.949**	
				[1.36, 6.395]	
Hispanic* high school					1.88
					[0.826, 4.28]
Hispanic* >high school					2.016
					[0.937, 4.335]

95% confidence intervals in brackets. *p < 0.05; **p < 0.01; ***p < 0.001.

## Discussion

We found that although mental health diagnosis rates for Hispanics were lower, more Hispanics reported a higher K6 score than their White counterparts, which may indicate a disproportionate and unmet need for mental healthcare services. Our findings are consistent with the literature showing that Hispanics face an unmet need for mental healthcare services that is more than double that of their White counterparts (6, 8, 9). Moreover, high psychological distress that does not manifest itself into proportionately high diagnosis rates can also mean that there is an underutilization of services by Hispanic respondents. This is also consistent with research showing that, even when services are readily accessible, Hispanics have lower mental health service utilization rates than Whites (6, 10, 13).

Among both Hispanics and Whites, K6 scores were negatively associated with household income. Our findings suggest that low psychological distress and good mental health may be positively associated with increases in socioeconomic position, which is consistent with findings in the available literature (6, 7). Moreover, research has also shown that foreign-born Hispanics are at significantly lower risk for psychiatric morbidity than US-born Whites (24), which is also consistent with our findings showing better K6 scores for foreign-born Hispanics than US-born Hispanics and US-Born Whites. Furthermore, research also suggests that acculturation among Hispanics is associated with an increase in the prevalence of psychiatric disorders and illicit drug use (20, 24). Although the mechanism through which acculturation affects mental health is understudied, researchers have posited that Hispanic culture may be protective and exposure to and adoption of some elements of U.S. culture may have detrimental effects on psychiatric morbidity (25).

Hispanic participants in our study reported lower rates of lifetime and current use of illicit substances than their White counterparts. This is consistent with the research showing that the prevalence of illicit drug use is lower among US Hispanics than Whites (35–37). While high-income Whites report slightly less illicit drug use than low-income Whites, the prevalence of lifetime illicit drug use is three times higher among Hispanics in the highest income stratum relative to those in the lowest. The drastic increase in illicit drug use for Hispanics indicates that increased income may be associated with potential risk factors for illicit drug use. However, studies suggest that foreign-born Hispanics are at significantly lower risk for illicit drug use than US-born Whites (25, 26), which is also consistent with our findings showing that nativity may be a protective factor for Hispanics. Hispanic and other ethnic identification has been associated with decreased illicit drug use (26). Other research has shown that close social networks and family ties are also a protective factor against illicit drug use among Hispanics (27). These studies may suggest that the lower illicit drug use among low-income Hispanics may be due to stronger social support among low-income, foreign-born Hispanics relative to their higher-income, US-born counterparts. This conclusion is supported by our multivariate results, which indicate that mental health disparities between Whites and Hispanics are largely explained after adjustment for income and nativity.

This study has several limitations. One is that we conducted secondary analyses of the AHS data, which limited our mental health outcomes and explanatory variables to those collected by AHS. Moreover, since AHS was a telephone survey, the sample may not have included those with only cell phones or those that do not have a phone. This limitation is particularly salient among lower income populations. The AHS data include sample weights that attempt to correct for the complex survey design and non-response bias, which may limit the impacts of the design on our study. A further limitation is that all measures are self-reported and thus subject to bias. The proportion of participants who self-reported as illicit drug users was small, which may have limited our statistical power to assess disparities across groups. Lastly, there were a large proportion of participants who did not provide household income information. Our analysis included only a subpopulation of those with complete information on all variables used, which may have been detrimental to our analysis. One consequence of the small prevalence of self-reported drug use was that the time frame we used in our multivariable model predicting drug use was very long (i.e., the outcome in Table 4 was lifetime illicit drug use). Lifetime drug use is a very gross variable and may not be indicative of current need for behavioral health services. Despite these limitations, the AHS is one of very few population-based studies to assess the health of the Arizona population. Little is known about health in Arizona, particularly beyond vital statistics data and data collected in large national surveys that include substantial state samples (e.g., the Behavioral Risk Factor Surveillance System). Thus, the large sample size, population-based design, inclusion of a large number of minority (i.e., Hispanic) participants, and measurement of a relative wealth of behavioral data are strengths of AHS and, in turn, this study.

In this study, we found evidence of mental health and healthcare disparities between Whites and Hispanics in Arizona. Despite similar prevalence of psychological distress, Hispanics were much less likely to have been diagnosed with a mental health condition. Furthermore, while Hispanics on average were less likely to report illicit drug use, the likelihood of illicit drug use among Hispanics greatly increased with income and among the US-born and the non-English language proficient. These disparities, combined with the rapid growth of the Hispanic population, suggest that developing culturally- and linguistically appropriate strategies to improve generally poor access and use of mental healthcare services among Hispanics is of critical public health importance. Our work should be a guide for future surveillance and intervention research on the complex relationship between race/ethnicity, socioeconomic status, mental health, and health care.

## Conflict of Interest Statement

The authors declare that the research was conducted in the absence of any commercial or financial relationships that could be construed as a potential conflict of interest.
